# Transfer function of Brazilian Portuguese oral vowels: a comparative acoustic analysis

**DOI:** 10.1016/S1808-8694(15)30518-8

**Published:** 2015-10-18

**Authors:** Maria Inês Rebelo Gonçalves, Paulo Augusto de Lima Pontes, Vanessa Pedrosa Vieira, Antônio Augusto de Lima Pontes, Daniella Curcio, Noemi Grigoletto De Biase

**Affiliations:** 1 Post-PhD, Adjunct Professor of Speech and Hearing Therapy, Federal University of São Paulo, São Paulo, SP, Brazil. Head of the Integrated Speech and Hearing Therapy Service, São Paulo Hospital, São Paulo, SP, Brazil. Associate Researcher at Instituto da Laringe - INLAR, São Paulo, Brazil; 2 Professor of the Otorhinolaryngology and Head and Neck Surgery Department, Federal University of São Paulo, São Paulo, SP, Brazil. Director at Instituto da Laringe - INLAR, São Paulo, Brazil; 3 Speech and Hearing Therapist; MSc in Sciences, Federal University of São Paulo; Voice Specialist; 4 MD, Otorhinolaryngologist at Instituto da Laringe - INLAR, São Paulo, Brazil; 5 Speech and Hearing Therapist. PhD in Sciences, Federal University of São Paulo, Brazil. Professor Instructor at the Morphology Department, Santa Casa School of Medical Sciences, São Paulo, Brazil; 6 Post-PhD, Associate Professor at the Department of Fundamentals, Speech and Hearing Therapy School, Catholic University of São Paulo, Brazil. Graduate Studies Advisor at the ENT and Head and Neck Surgery Department, Federal University of São Paulo, São Paulo, Brazil. Instituto da Laringe

**Keywords:** speech acoustics, sound spectrography, phonetics, voice

## Abstract

The vocal tract transfers its characteristics onto the sounds produced at the glottis, depending on its tridimensional configuration.

**Aim:**

this study aims to determine which of the seven oral vowels in Brazilian Portuguese is acoustically less impacted by changes to the vocal tract.

**Materials and method:**

this is a cross-sectional prospective study. Twenty-three males and 23 females with ages ranging between 20 and 45 years (mean values of 28.95 and 29.79 years respectively) were enrolled in the study; none had voice complaints and their voices were normal under perceptive-auditory evaluation. Three-hundred and twenty-two sustained vocal emissions were digitized and acoustically analyzed by three computer programs combined. Results were compared against the distribution of resonance frequencies in a straight tube with one end sealed.

**Results:**

statistical analysis showed that vowel /ε/ was significantly different when compared to the other vowels, with higher mean harmonic values and lower standard deviation for both genders.

**Conclusion:**

in BrazilianP ortuguese, vowel /ε/ is less impacted by changes to the vocal tract and is significantly less attenuated in both genders. The inclusion of this vowel in voice assessment standard protocols may contribute to improve the quality of the information obtained as a result of quantitative spectrographic and acoustic tests.

## INTRODUCTION

The geometry of the vocal tract in adults is similar to a straight tube sealed on one of its ends; additionally, it averages 17 cm in length and its three first peaks of resonance range at about 500, 1500 and 2500Hz^1^, ^2^, ^3^ respectively.

The vocal tract transfers its characteristics to the sounds produced at the glottis in accordance with its tridimensional configuration, which results from the positioning of its component structures. Such transfer, also referred to as transfer factor, changes the intensity of harmonics, as a consequence of the resonance phenomenon. Harmonic frequencies coinciding with vocal tract resonance frequencies undergo minor changes and are called formants, whereas all others have their intensities reduced (or not amplified)^2^,^4^. Formants vary depending on the tridimensional arrangement of the vocal tract; the first three formants are fundamental for vowel acoustic identity.

Standardized speech evaluation protocols preferentially use vowels /a/, /i/, /u/ as they are acoustically very different from each other, thus favoring the observation of possible disturbances in the various parts of the spectrum against the corresponding configurations of the vocal tract.

Observation of the vocal tract configuration during vowel emission shows a more uniform tubular cross section during the phonation of vowel /ε/. Clinical observation of spectrographic data of Brazilian Portuguese vowels has generally shown higher intensity harmonics in emissions of vowel/ε/, possibly indicating lesser modifications in the shape of the vocal tract in comparison to when other oral vowels are uttered. Thus, vowel /ε/ is the closest in similarity to a straight tube configuration and possibly the one of highest intensity, and consequently better definition of harmonics. Still looking at the visual configuration of the vocal tract, vowel /u/, as the one with the most significant changes to the vocal tract, introduces more obstacles to the passage of sound and is consequently characterized by reduced intensity and poorer definition of harmonics.

Therefore, it is important to objectively identify the vowel that allows for the most accurate association of laryngoscopic images and acoustic data.

This study aims to compare the acoustic identity of the seven oral vowels in Brazilian Portuguese and set apart the one that is least impacted by changes to the vocal tract when compared to a straight tube sealed on one of its ends.

## MATERIALS AND METHOD

The seven oral vowels of Brazilian Portuguese (/a/, /ε/, /e/, /i/, /É/, /o/ e /u/) produced by 23 males and 23 females aged between 20 and 45 years (mean values of 28.95 and 29.79 years respectively) were recorded. One recording was made for each individual vowel as emitted by each study subject, adding up to 322 recordings.

Two speech and hearing therapists and two otorhinolaryngologists selected the subjects enrolled in the study; enrollment criteria were absence of voice-related complaints and normal voice quality under perceptive-auditory evaluation.

Subjects were asked to emit each vowel in a sustained fashion at their usual frequency and intensity. Recordings were carried out in a soundproof booth; subjects' lips were kept 15 centimeters away from a unidirectional SHURE SM58 microphone. Sound digitization was performed using Macintosh's SoundScope software program. After recording, all 322 sound waves were equalized at 4 volts in amplitude. Samples were standardized in 5-second segments in the mid portion of the sound wave.

Sound waves were then converted to .wav format using software program Wave Converter 1.5. Quantitative data sets were obtained through the combined use of three computer programs: Vocal Assessment, Screen Size Capture and Carnoy 2.0, as follows:

Step 1. Vocal Assessment - used to select the 5-second segment picked for analysis and to separate noise harmonics, leaving only harmonic peaks.

Step 2. Carnoy 2.0 - used to quantify harmonic peaks from image files generated from the noise-free graphs.

Intensity values for all harmonics were recorded for each individual sample. Mean values and standard deviations were calculated so as to allow comparisons intra and inter individuals for harmonic intensity of each vowel.

Software program SoundScope was used to analyze harmonic frequencies and calculate the f0 values for each vowel and individual; such values were then multiplied by the number of the harmonic of greater intensity in the region corresponding to the three first formants of each vowel, so as to determine the frequency of each formant. For example, if f0 were equal to 100 Hz and the fifth harmonic were the one of greater intensity in the first formant region, than its frequency would be 500 Hz. The mean frequency values of the three harmonics of greater intensity were obtained (Peterson, 1959) for both the male and the female groups. These mean values, representing the regions of the three formants, were compared to the resonance distribution in a straight tube sealed on one of its ends, in which the frequency of the other formants are whole odd multiples of the first. In the specific case of a tube with approximately 17 cm in length, these frequencies amount to approximately 500, 1500 and 2500 Hz. Thus, we considered the vowel whose vocal tract assumes the closest to a tubular shape and that would cause the least change to glottal sounds, i.e., whose mean values are closer to these three values. Such vowel is highlighted in bold type on the table describing the harmonic frequency values.

Considering the nature of the studied variables, the Friedman test (non-parametric) was carried out to find whether the mean values were statistically different (p<0.001). As statistical significance was found, then at least one of the seven deviations was different from the remaining ones. Thus, we also used Student's T-test for paired data (parametric test) to determine the pairs with statistically significant difference, and the Wilcoxon signed-rank test (non-parametric test) to compare the ranked distribution (Rosner, 1986). Values with a significance level of 0.050 (5%) were marked with an asterisk.

## RESULTS

Results can be seen on Table 1, Table 2, Table 3. Table 1 shows the mean harmonic intensity values for each vowel and their respective standard deviations, as obtained for each gender. Table 2 shows the statistical data used to compare harmonic intensities pair by pair. Table 3 shows the frequency values for the first three formants of each vowel separated for gender.

**Table 1:** Mean harmonic intensity values (db) and their respective standard deviations for each vowel as produced by each gender group.

	FEMALES		MALES	
Vowel	X	DP	X	DP
/a/	42,92	9,48	36,91	11,02
/ε/	45,04	6,88	39,37	9,27
/e/	43,88	8,32	38,85	10,3
/i/	41,49	10,7	36,73	10,97
/⊃/	39,94	11,56	36,33	11,63
/o/	36,5	13,25	33,84	12,91
/u/	35,29	13,56	32,78	13,02

**Table 2:** p values in the paired analysis of vowel harmonic intensity mean values and respective standard deviations for each gender group.

	FEMALES		MALES	
Vowel pairs	P		P	
	X	DP	X	DP
/a/×/ε/	0,005^*^	0,001^*^	0,007^*^	0,001^*^
/a/×/e/	0,139	0,010^*^	0,013^*^	0,062
/a/×/i/	0,148	0,031^*^	0,832	0,913
/a/×/⊃/	0,003^*^	0,001^*^	0,519	0,076
/a/×/o/	0,001^*^	0,001^*^	0,001^*^	0,001^*^
/a/×/u/	0,001^*^	0,001^*^	0,001^*^	0,001^*^
/ε/×/e/	0,052	0,001^*^	0,349	0,007^*^
/ε/×/i/	0,001^*^	0,001^*^	0,001^*^	0,001^*^
/ε/×/⊃/	0,001^*^	0,001^*^	0,001^*^	0,001^*^
/ε/×/o/	0,001^*^	0,001^*^	0,001^*^	0,001^*^
/ε/×/u/	0,001^*^	0,001^*^	0,001^*^	0,001^*^
/e/×/i/	0,015^*^	0,001^*^	0,011^*^	0,085
/e/×/⊃/	0,001^*^	0,001^*^	0,006^*^	0,001^*^
/e/×/o/	0,001^*^	0,001^*^	0,001^*^	0,001^*^
/e/×/u/	0,001^*^	0,001^*^	0,001^*^	0,001^*^
/i/×/⊃/	0,170	0,064	0,614	0,093
/i/×/o/	0,001^*^	0,001^*^	0,003^*^	0,001^*^
/i/×/u/	0,001^*^	0,001^*^	0,001^*^	0,001^*^
/⊃/×/o/	0,001^*^	0,001^*^	0,004^*^	0,001^*^
/⊃/×/u/	0,001^*^	0,001^*^	0,001^*^	0,001^*^
/o/x/u/	0,168	0,266	0,194	0,547

Student's t-test for paired data. Statistical significance is marked with an asterisk for p<0.050.

**Table 3:** Mean harmonic frequency values for the first three formants (F1, F2, F3) in Hz, for each vowel and gender group.

VOWELS	FEMALES			MALES		
	F1	F2	F3	F1	F2	F3
/a/	1002,90	1549,95	2959,70	753,87	1278,70	2483,44
/ε/	672,45	2242,93	3018,60	588,44	1745,11	2566,00
/e/	437,03	2429,76	3087,09	406,63	1955,60	2540,33
/i/	361,90	2583,89	3378,14	297,80	2150,85	2925,14
/⊃/	715,34	1073,27	2981,69	580,15	947,25	2525,52
/o/	444,89	914,26	2899,80	411,62	832,84	2376,13
/u/	461,82	763,41	2902,55	345,27	799,51	2351,50

## DISCUSSION

Only the Brazilian Portuguese oral vowels were studied. Two are the reasons for that, as follows: (1) while performing vocal analysis and laryngological evaluation, nasal vowels are usually not emitted, unless the soft palate is being analyzed; in clinical speech and hearing assessment, oral vowels /a/, /i/ and /u/ are the ones more commonly used; in laryngological assessment, usually vowels /a/ or /ε/ (for modal register) and /i/ (for high pitch or falsetto emissions) are more frequently used, although there is no consensus in the professional community. (2) The second reason has to do with the spectrographic analysis of nasal vowels, made more difficult by the presence of additional formants of reduced intensity; besides, nasal vowels generate a greater number of identification errors than oral vowels^5^.

Energy input of the samples was equalized at about 4 volts in order to standardize the intensity output levels, as subjects were requested to emit vowels in their usual and comfortable frequency and intensity.

Vowel /e/ had greater mean harmonic intensity values with lesser standard deviation (Table 1). When looking at paired mean values (Table 2), we observed that vowel /ε/ was significantly different from all others, except for vowel /e/, for both genders. Paired standard deviations also showed statistically significant differences between vowel /ε/ and all other vowels. Thus, we may state that, additionally from presenting greater mean values and lower standard deviations in harmonic intensity, vowel /ε/ was significantly different from all other vowels (except for the mean values found for vowel /e/) for both genders. The similarity between vowels /ε/ and /e/ might be due to the fact that both have similar vocal tract configurations. Nonetheless, on vowel /ε/ the tongue is located at a lower position than when vowel /e/ is uttered, that is, it is less constricted and there consequently are fewer obstacles to the passing of sound energy. Therefore, although the difference between the values found for both vowels is not statistically significant, they were still greater for both genders on the emissions of vowel /ε/ and the p value (0.052) indicates a trend towards significance.

Greater mean values and lower standard deviations are related to lesser vocal tract attenuation^4^,^6^,^7^. Such fact implies that, in the vocal tract course, the position of the resonators in the emissions of vowel /ε/ allows the passing of more energy with less interference upon the sound produced by vocal fold vibration, indicating a transfer factor whose frequencies coincide with the tract resonance frequencies without deformation. More specifically, they indicate that the positioning of the structures allows close-to-free flow through the vocal tract, as observed in vowel /ε/. We should however bear in mind that wave amplitude is essentially a measurement of its size, an entity independent from wave frequency. Therefore, sound energy is present in each harmonic, but the amplitude of each harmonic is impacted by the filter function and the amplitude of the harmonic specific to the glottal source^4^.

According to Hermann^8^, formant frequencies are, in reality, the ones in which the supralaryngeal filter allows the passing of the greater amount of energy. This is due to the minimal interference the vocal tract has had upon the sound produced by vocal fold vibration, once vocal tract changes tend to reduce the acoustic information of the sound generated in the larynx. Vowel /ε/ in Brazilian Portuguese is produced with lesser harmonic attenuation, thus better representing the spectrum of the glottal source. Our findings are in agreement with those of other authors^9^, in that during the articulation of vowel /ε/ the vocal tract works acoustically as a tube sealed on one of its ends, based on the findings of MRI three-dimensional reconstructions of the vocal tract.

The frequencies of the three first formants (Table 3) showed that vowel /ε/ had values closer to those of the transfer function of a neutral vowel, i.e., 500, 1500 and 2500 Hz, than the other vowels in males. The same was observed in relation to females, but by a larger difference. Measurements were tougher in this group, as women f0 of higher pitch and consequently more spaced, fewer harmonics throughout the spectrum. Nonetheless, in spite of the greater spaces between values, the proportion between the three formants was also kept closer for vowel /ε/ among females.

However, other authors studying the sounds of other languages have concluded that vowel /æ/ is the closest to the vocal tract transfer function, i.e., to formant frequencies F1, F2 and F3 closer to 500, 1500 and 2500Hz^10^. Other studies have described the English vowel schwa (/ε/) as the one closest to the transfer function referred to as the neutral vowel 11-15. Thus, we may suggest the inclusion of vowel /ε/ in standardized protocols for vocal assessment in Brazil.

## CONCLUSION

After observing the acoustic characteristics of the emissions of Brazilian Portuguese vowels, we found that the distribution of the first three formants of vowel /ε/ is the closest to the resonance frequencies seen in a straight tube sealed on one of its ends and, consequently, to the neutral vowel, as it is the least impacted by vocal tract changes for both genders.
